# IntroVerse: a comprehensive database of introns across human tissues

**DOI:** 10.1093/nar/gkac1056

**Published:** 2022-11-18

**Authors:** Sonia García-Ruiz, Emil K Gustavsson, David Zhang, Regina H Reynolds, Zhongbo Chen, Aine Fairbrother-Browne, Ana Luisa Gil-Martínez, Juan A Botia, Leonardo Collado-Torres, Mina Ryten

**Affiliations:** Department of Genetics and Genomic Medicine Research & Teaching, UCL GOS Institute of Child Health, London, WC1N 1EH, UK; Department of Neurodegenerative Disease, Queen Square Institute of Neurology, UCL, London WC1N 3BG, UK; Department of Genetics and Genomic Medicine Research & Teaching, UCL GOS Institute of Child Health, London, WC1N 1EH, UK; Department of Neurodegenerative Disease, Queen Square Institute of Neurology, UCL, London WC1N 3BG, UK; Department of Genetics and Genomic Medicine Research & Teaching, UCL GOS Institute of Child Health, London, WC1N 1EH, UK; Department of Neurodegenerative Disease, Queen Square Institute of Neurology, UCL, London WC1N 3BG, UK; Department of Genetics and Genomic Medicine Research & Teaching, UCL GOS Institute of Child Health, London, WC1N 1EH, UK; Department of Neurodegenerative Disease, Queen Square Institute of Neurology, UCL, London WC1N 3BG, UK; Department of Genetics and Genomic Medicine Research & Teaching, UCL GOS Institute of Child Health, London, WC1N 1EH, UK; Department of Neurodegenerative Disease, Queen Square Institute of Neurology, UCL, London WC1N 3BG, UK; Department of Genetics and Genomic Medicine Research & Teaching, UCL GOS Institute of Child Health, London, WC1N 1EH, UK; Department of Neurodegenerative Disease, Queen Square Institute of Neurology, UCL, London WC1N 3BG, UK; Department of Medical and Molecular Genetics, School of Basic and Medical Biosciences, King's College London, London, WC2R 2LS, UK; Department of Neurodegenerative Disease, Queen Square Institute of Neurology, UCL, London WC1N 3BG, UK; Department of Information and Communications Engineering Faculty of Informatics, Espinardo Campus, University of Murcia, Murcia, 30100, Spain; Department of Information and Communications Engineering Faculty of Informatics, Espinardo Campus, University of Murcia, Murcia, 30100, Spain; Lieber Institute for Brain Development, Baltimore, MD 21205, USA; Department of Genetics and Genomic Medicine Research & Teaching, UCL GOS Institute of Child Health, London, WC1N 1EH, UK; Department of Neurodegenerative Disease, Queen Square Institute of Neurology, UCL, London WC1N 3BG, UK; NIHR Great Ormond Street Hospital Biomedical Research Centre, University College London, London, WC1N 1EH, UK

## Abstract

Dysregulation of RNA splicing contributes to both rare and complex diseases. RNA-sequencing data from human tissues has shown that this process can be inaccurate, resulting in the presence of novel introns detected at low frequency across samples and within an individual. To enable the full spectrum of intron use to be explored, we have developed IntroVerse, which offers an extensive catalogue on the splicing of 332,571 annotated introns and a linked set of 4,679,474 novel junctions covering 32,669 different genes. This dataset has been generated through the analysis of 17,510 human control RNA samples from 54 tissues provided by the Genotype-Tissue Expression Consortium. IntroVerse has two unique features: (i) it provides a complete catalogue of novel junctions and (ii) each novel junction has been assigned to a specific annotated intron. This unique, hierarchical structure offers multiple uses, including the identification of novel transcripts from known genes and their tissue-specific usage, and the assessment of background splicing noise for introns thought to be mis-spliced in disease states. IntroVerse provides a user-friendly web interface and is freely available at https://rytenlab.com/browser/app/introverse.

## INTRODUCTION

Since the discovery of introns in 1977 (1,2), there has been increasing interest in splicing and, in particular, in alternative splicing as a means of generating transcriptomic diversity. In humans, it is now recognised that nearly all multi-exon genes have multiple isoforms (3,4) and that dysregulation of splicing contributes to both rare and complex disease (5–7). Many of these insights have been gained using short-read RNA-sequencing data (8), which enables high throughput novel intron discovery by identifying reads which map with a gapped alignment to the genome, termed junction reads. To date, most RNA-sequencing splicing analyses have focused on identifying functionally important transcripts, their exons and usage, with an increasing interest in disease-specific applications as exemplified by RJunBase (9) (that offers an extended classification of splice junctions from non-cancerous and cancerous human samples). These priorities are reflected in existing splicing databases, which commonly filter splicing events based on their frequency, such that only a subset of introns thought to be part of stable transcripts are visible to the user (10–13) or do not provide a freely-available user-friendly web tool to browse and explore all detected novel junctions and their implied introns (14,15).

However, the increasing depth and availability of large quantities of publicly accessible RNA-sequencing data have made clear that splicing errors are common (16–19), resulting in the consistent detection of partially-unannotated junction reads at low frequency across samples and within individuals (19). Quantifying these errors, differentiating them from splicing events due to the presence of novel rare transcripts and identifying patterns in splicing variation have become increasingly important with the recognition of the role of splicing in ageing (DOI:10.1101/2022.03.14.484341), the use of RNA-sequencing data to improve diagnostics (20) and the use of long-read RNA-sequencing data for discovery of rare transcripts (21). Despite this interest, there are no existing databases that allow the full spectrum of intron use to be explored.

To bridge this gap, we created IntroVerse, which currently offers an extensive catalogue on the usage of 332,571 annotated introns and a linked set of 4,679,474 novel junctions and their implied novel introns covering 32,669 different genes. This data set was generated through the analysis of 17,510 human control RNA samples across 54 tissues provided by the Genotype-Tissue Expression Consortium v8 (22) and processed by recount3 (23). IntroVerse has two unique features: (i) it provides a complete catalogue of carefully processed and quality-controlled annotated introns and (ii) each novel junction (as implied by a novel split read) has been assigned to a specific annotated intron. The latter is key to the creation of a hierarchical database structure which allows the user to start by browsing a gene or genomic position of interest and end with an exploration of the entire catalogue of alternative splicing events. To enable users to prioritise annotated introns and novel junctions of greatest interest, we also provide complementary information on key parameters including: measures of splicing accuracy at each annotated intron, the percentage of individuals in which each splicing event has been observed, 5’ and 3’ sequence strength (24), sequence conservation (25) across primates in proximal intronic regions, sequence constraint (26) across humans in proximal intronic regions and mean read coverage. Furthermore, IntroVerse offers the user the opportunity to visualise novel junctions and their frequency within the main transcript structure, with the resulting plots downloadable in SVG format.

IntroVerse has common use cases relevant to a range of user groups which include: (i) the assessment of background splicing noise for introns thought to be mis-spliced in disease states so aiding diagnosis of rare pathogenic splicing mutations and (ii) the identification of novel transcripts of known genes and their tissue-specific usage. Focusing on the latter, we note that in a single tissue, frontal cortex, we detected 2,048 novel junctions which are used in > 80% of independent samples (*N* = 167) and which correspond to 1,420 genes suggesting the existence of incomplete annotation at these sites. Importantly, we found that the discovery of such sites was only weakly correlated with read coverage (*R*^2^ = 3.7e-2, *P-*value < 2e−16, Supplementary Figure S1). Thus, IntroVerse enables the efficient identification of genes for which existing transcriptome annotation is incomplete, providing key information to direct targeted analyses.

IntroVerse does not require registration or login for access, and it is freely available for research use and data download from multiple devices, screen sizes and browsers at https://rytenlab.com/browser/app/introverse. IntroVerse is also available in docker format at https://hub.docker.com/r/soniaruiz/introverse.

## MATERIALS AND METHODS

### GTEx v8 data collection and processing of split read data

**Figure 1.**
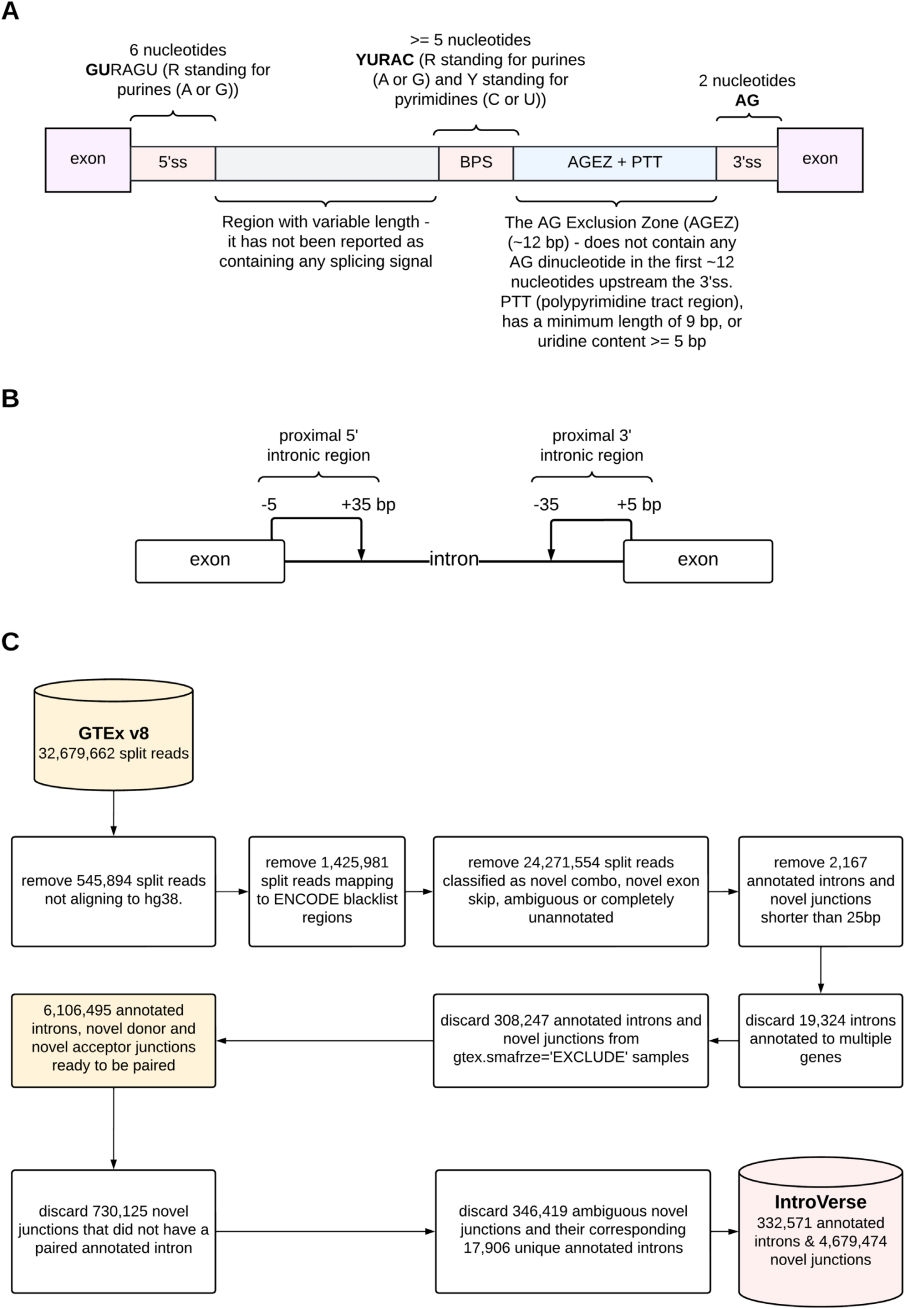
Overview of the quality-control steps applied to the original dataset of split reads provided by GTEx v8 and processed by recount3. (**A**) Length of the canonical splicing signals. All split reads considered were at least 25 bp long. (**B**) Length of the proximal intronic regions that were considered for the calculation of the mean conservation score across primates and mean constraint values across humans of the overlapping sequences. (**C**) Flow chart containing the complete quality-control process applied to the original dataset of split reads provided by GTEx v8 and processed by recount3 before the construction of IntroVerse.

**Figure 2.**
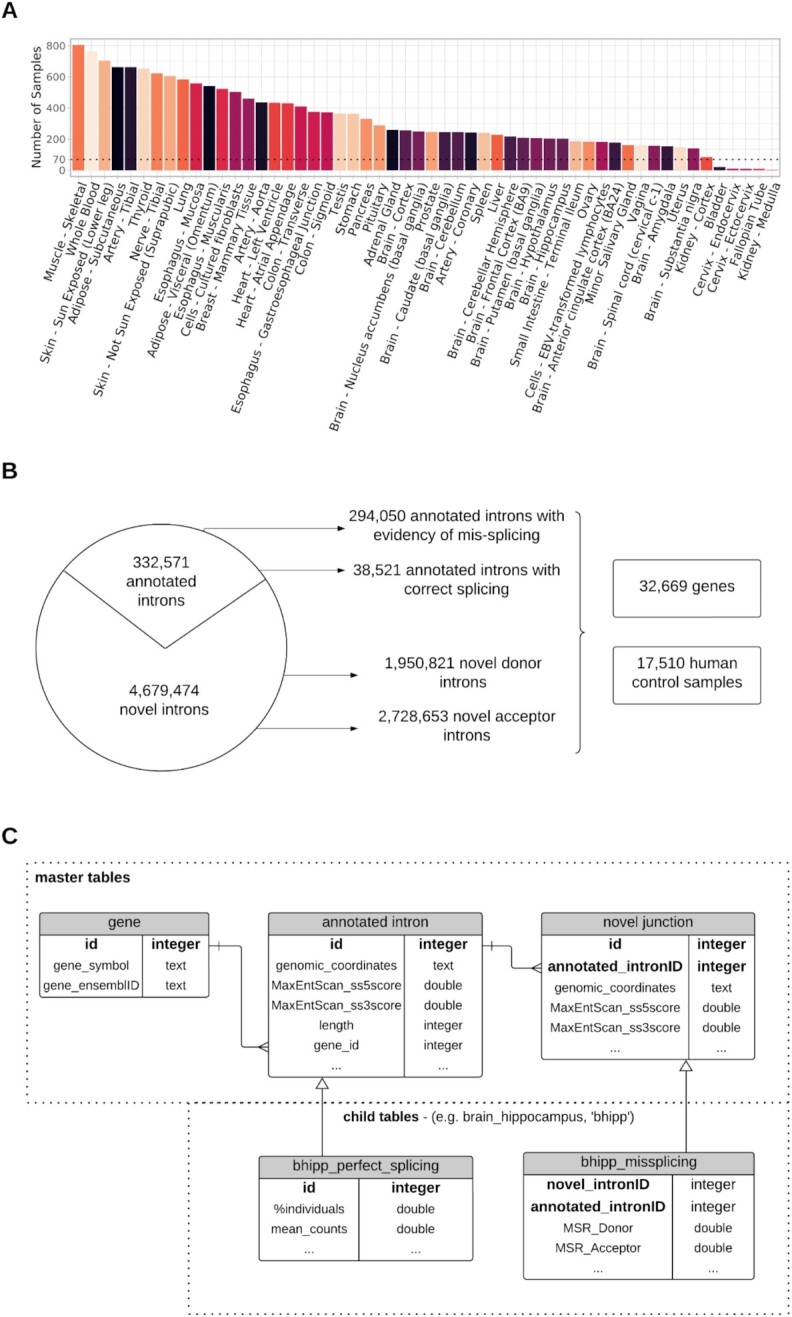
Data statistics and SQL Schema designed for IntroVerse. (**A**) Sample count by tissue. Each bar represents the number of samples per body site considered in IntroVerse. (**B**) Database summary statistics. (**C**) SQL schema design for IntroVerse. The table *‘annotated intron’* corresponds to the table *‘intron’* in the SQLite schema, and the table *‘novel junction’* refers to the table *‘novel’* in the SQLite schema.

IntroVerse is based on RNA-sequencing data generated by the GTEx v8 (22) project. As such it includes data on 17,510 samples across 948 post-mortem donors and 54 tissues after accounting for the exclusion of samples that did not meet the minimum GTEx quality control criteria (gtex.smafrze = ‘EXCLUDE’; *N* = 1,571) (Figure 1, Figure 2). RNA-sequencing data from the project, which used the Illumina TruSeq library construction protocol (non-stranded 75 base pairs (bp) long reads, polyA+ selection), was processed by recount3 using Monorail (23). The latter implements STAR (27) to align reads to the human genome (hg38/GRCh38) and summarise split reads (those mapping to the genome with a gapped alignment), and Megadepth (28) version 1.0.3 to analyse the BAM file outputs generated by STAR. We downloaded all split read data corresponding to GTEx v8 samples using the R package recount3 version 1.6.0.

To identify split reads that were most likely to be generated through the activity of spliceosomes, we removed all reads whose implied intron length (defined by the gap in alignment to the genome) was less than 25 bp, the minimum length required for all essential splicing signals (29) (Figure 1). In order to increase the confidence in the spliceosome origin of these split reads, we discarded the reads located within unlocalized and unplaced (i.e. random) sequences, due to their inability to be located within the hg38 chromosomes 1–22 (chr1–chr22), X (chrX), Y (chrY) with a specific order and orientation. We also discarded all split reads that overlapped with any regions published within the ENCODE Blacklist (30) (Figure 1) as this list contains the DNA regions (hg38) where assembly had been difficult and, thus, their removal result in an essential quality measure.

The split reads meeting these initial quality-control criteria were then annotated to the Ensembl v105 reference transcriptome by using the junction_annot() function made available through the Bioconductor R package dasper (DOI:10.1101/2021.03.29.437534) version 1.4.3. Depending on the genomic coordinates of the split reads, these were classified by dasper into seven different junction categories: annotated, novel donor, novel acceptor, novel combo, novel exon skip, ambiguous and completely unannotated. Due to their unique ability to be linked to an annotated intron and, therefore, to a gene in annotation, we only considered reads classified as (i) *‘annotated’* junction reads, defined as split reads for which both ends of the implied intron precisely matched an intron within annotation, (ii) *‘novel donor’* junction reads, defined as split reads for which only the acceptor site precisely matched an intron-exon boundary within annotation and (iii) *‘novel acceptor’* junction reads, defined as split reads for which only the donor site precisely matched an exon-intron boundary within the annotation (Figure 1C). These three types of split read, henceforth referred to as annotated introns, novel donor and novel acceptor junctions, were incorporated into IntroVerse. The latter two categories, collectively referred to as novel junctions, implied the usage of novel introns.

### Identification of mis-splicing events and their association to annotated introns

By default, novel junctions meeting the criteria defined in *GTEx v8 data collection and processing ofsplit read data*, were considered to be products of mis-splicing events generated through the inaccurate excision of annotated introns (defined by Ensembl v105). While this definition of mis-splicing assumes that current transcriptome annotation is complete (which we recognise is not the case), it is important to note that the addition of novel introns into annotation has been plateauing for some time (14) making this a reasonable working assumption.

**Figure 3.**
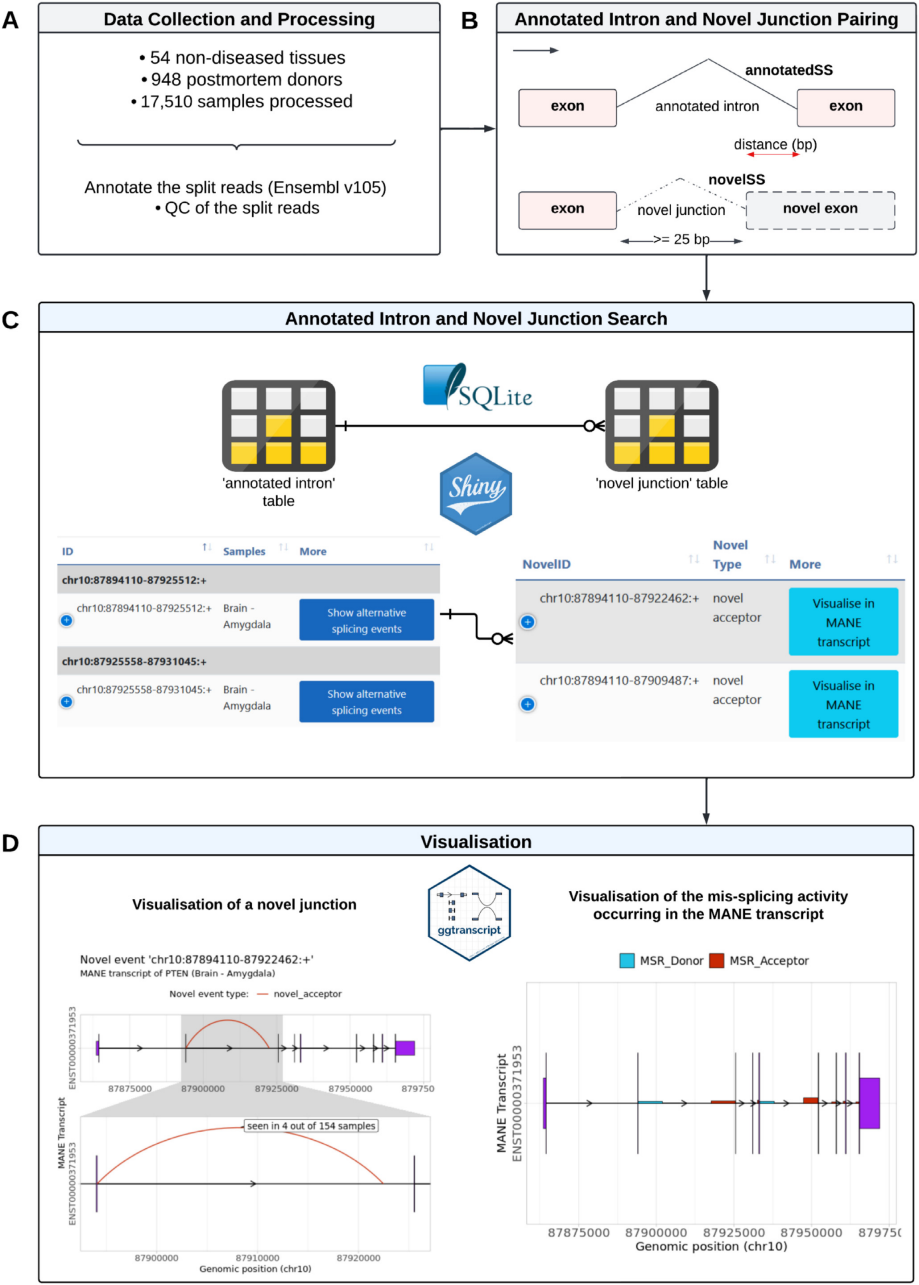
Overall design and construction of IntroVerse. (**A**) Summary of data collection and processing. (**B**) Summary of the process used for annotated intron and novel junction pairing. ‘AnnotatedSS’ refers to annotated splice site and ‘novelSS’ refers to novel splice site. (**C**) Summary of the cardinality between the annotated introns and novel junctions in IntroVerse. (**D**) Visualisation of a novel junction and the mis-splicing activity occurring within the MANE transcript of a selected gene using ggtranscript (35).

Next, to enable the identification of mis-splicing events, we started by assigning each novel donor and acceptor junction to a known annotated intron through sharing of their single annotated splice site (Figure 3B). To increase confidence in junction pairing, we ensured that each novel junction was only paired to an annotated intron if there was also evidence for use of that specific annotated intron in every sample in which the novel junction was found, as defined by the presence of ≥ 1 annotated junction read in each sample. In total, 730,125 novel donor and acceptor junctions were discarded from this project as there was not any compatible annotated intron meeting the aforementioned criteria to pair them with among the set of samples studied.

Since we considered mis-splicing to be the result of the inaccurate excision of annotated introns, a novel junction could only be the result of the mis-splicing of a single unique intron, regardless of the sample or body site in which the novel event had been found. Consequently, all novel donor and acceptor junctions able to be paired to multiple annotated introns within a sample or across samples, were considered ambiguous and discarded from this project. This resulted in the discard of 346,419 ambiguous novel junctions and 17,906 annotated introns that were uniquely parenting them, with none of them included in IntroVerse. In total, 1,094,450 annotated introns and novel junctions were excluded from IntroVerse (Figure 1).

### Quantifying the levels of mis-splicing at an annotated intron using the mis-splicing ratio measure

To determine the mis-splicing rate for any annotated intron across samples of a given tissue, we generated a measure, which we termed the mis-splicing ratio ($MSR$). Two $MSR$ values were calculated for each annotated intron to provide a measure of the frequency of mis-splicing at the donor ($MS{R_D}$) and acceptor splice sites ($MS{R_A}$) separately.

The $MS{R_D}$ and $MS{R_A}$ measures for a given annotated intron were calculated by first summing together all novel donor and novel acceptor junction read counts respectively that were attached to the annotated intron across all samples of the tissue, and then dividing that value by the sum of all annotated intron and novel junction read counts across the same set of samples. Focusing on the $MS{R_D}$ measure, this can be formally described as below:

$$\begin{equation*}MSR_D^X = \frac{{\mathop \sum \nolimits_{i = 1}^N {j_i}}}{{\mathop \sum \nolimits_{i = 1}^N {j_i} + \mathop \sum \nolimits_{i = 1}^N {s_i}}}\end{equation*}$$

Let $j$ denote the total number of novel donor junction reads assigned to the annotated intron $X$ within one sample of our study set. Let $s$ denote the total number of annotated junction reads for the same intron, $X$, within the same sample of our study set. Let $N$ denote the total number of samples studied within our study set.

We then mirrored the formula above to produce the $MS{R_A}$ measure.

Both $MS{R_D}$ and $MS{R_A}$ values were calculated per annotated intron across the samples of each tissue, and ranged between 0 and 1. Whereas 0 value represented accurate splicing at the donor or acceptor site of the annotated intron of interest as compared to the reference transcriptome Ensembl v105, 1 value represented complete mis-splicing of the splice site. This adaptation of the method for normalisation of library sizes (31) accounted for variability in expression and read depth across different genes and introns, enabling the comparison of mis-splicing activity across introns.

It is important to note that both $MS{R_D}$ and $MS{R_A}$ scores were only calculated when there was evidence of the annotated intron being detected within the tissue as defined by the presence of ≥ 1 supporting split reads. Given that some transcripts are not expressed in all tissues, the corresponding annotated introns would not be detected, and so the $MS{R_D}$ and $MS{R_A}$ values were not calculated. In fact, the median number of annotated introns across tissues as stored on IntroVerse corresponds to 239,839 and ranges from 187,551 to 279,156 annotated introns (Supplementary Table S1).

### Database generation

All annotated introns, and their related novel donor and acceptor junctions, were classified by tissue and stored within tables in a relational database that was named IntroVerse. To build this database, we used the SQLite R package version 2.2.7 (Figure 3C). All annotated introns in all tissues were stored within a master table called *‘intron’*, whereas all novel donor and novel acceptor junctions were stored together within another master table called *‘novel’*. These two master tables were related with a cardinality of 1:*N*, meaning that each annotated intron could be paired with multiple novel junctions, whereas each novel junction could only be paired with a single, unique annotated intron. An additional master table was created to store information about all genes from which the annotated introns originated. Two child tables were then generated per GTEx tissue and contained: (i) all novel junctions found across samples of each tissue and paired with an intron in annotation and (ii) all annotated introns that showed accurate splicing activity (defined by Ensembl v105) across the samples of the studied tissue (Figure 2C).

Multiple intron- and gene-level features were incorporated as columns to the master tables of the IntroVerse database:

- **Intron-level properties:**
  - The intron length (in base pairs).
  - The strength of the 5’ or 3’ splicing signals as generated by the MaxEntScan (24) score (MES) algorithm. This method uses probabilistic models of sequence motifs involved in RNA splicing and their adjacent positions to score the 9 bp sequence at the 5’ splice site and the 23 bp sequence at the 3’ splice site. The higher the maximum entropy score assigned to a given sequence, the more closely the sequence evaluated corresponds to an annotated splice site.
  - The mean inter-species conservation (25) score across primates assigned to the 5’ and 3’ proximal exon-intron junction regions. The 5’ proximal region was defined as 5 bp upstream of the 5’ splice site to 35 bp into the downstream intron. The 3’ proximal region was defined as 35 bp upstream of the 3’ splice site to 5 bp into the downstream exon (Figure 1B). This score was calculated by obtaining the mean of the *phastCons20* scores (http://hgdownload.cse.ucsc.edu/goldenPath/hg38/phastCons20way/) assigned to each proximal intronic region and represents the probability of negative selection based on the number of substitutions occurring during evolution across primates. The mean *phastCons20* score ranges between 0 and 1, with a value of 0 meaning that the sequence is poorly conserved and 1 that the sequence is highly conserved.
  - The mean context-dependent tolerance score (CDTS) (26) assigned to the 5’ and 3’ proximal exon-intron junction regions (Figure 1B). The CDTS score provides a measure of sequence constraint across individuals. This score ranges between -1 and 1, with negative values indicating regions of the human genome which have the lowest sequence variation across individuals.
  - The maximum Transcript Support Level (TSL) assigned to the set of transcripts in which the annotated intron appears. This field can present one of the following values: 1, 2, 3, 4, 5 and tslNA. An intron with a TSL = 1 value means that it has been found within a set of transcripts whose model presents the highest support available. On the contrary, an intron with a TSL = 5 value means that it has been found within a set of transcripts with a poorly supported model structure. TSL = tslNA, means that none of the transcript structures in which the intron has been found is analysed (https://www.ensembl.org/info/genome/genebuild/transcript_quality_tags.html).
  - Whether the annotated intron is likely to be spliced out from the pre-mRNA molecule by the major (column ‘u2_intron = T’) or minor spliceosome assembly (column ‘u12_intron = T’). This data was obtained from The Intron Annotation and Orthology Database (IAOD) (32). When these two columns are set to a FALSE value, it represents an annotated intron that was not categorised within the IAOD database.
- **Gene-level properties**. It has been reported that the number of introns within a gene or its level of expression can affect the fitness cost of splicing errors (33). With this in mind, some gene-level properties have been incorporated into IntroVerse.
  - The gene length (in base pairs).
  - The number of annotated transcripts assigned to the gene based on Ensembl v105.
  - The median gene expression level (TPM) across all samples from each tissue and calculated using the getTPM()function from the R recount Bioconductor package version 1.22.0.
  - The percentage of protein-coding transcripts in which each annotated intron was found. This percentage can be unreliable when a gene has many transcripts with low TSL values.
  - Yes/no boolean value to confirm whether the intron of interest forms part of a Matched Annotation from NCBI and EMBL-EBI (MANE) (34) Select transcript.

The columns of the child tables contain information about the splicing activity of the annotated introns and novel junctions detected in each tissue:

- **Annotated introns:**
  - The rate of mis-splicing at donor and acceptor splice sites of the annotated intron as calculated by the $MS{R_D}$ and $MS{R_A}$ measures, which range between 0 and 1. Focusing on the $MS{R_D}$ measure, a $MS{R_D} = 0$ value represents accurate splicing at the 5’ splice site of the intron across all samples of the tissue studied, whereas a $MS{R_D} = 1$ value would represent complete mis-splicing at that splice site. It is worth noting that the $MSR$ metrics do not convey information about the certainty in the measure of mis-splicing, since annotated introns with low or high read coverage can generate similar $MSR$ values. With this in mind, the reader should consider additional metrics provided by IntroVerse such as the percentage of samples in which the intron was found and its mean coverage.
  - The percentage of samples of a given tissue in which the annotated intron was used, as defined by ≥ 1 annotated read count in the sample.
  - The mean number of annotated reads counts assigned to the annotated intron across all the samples.
- **Novel junctions**:
  - The intron length in base pairs implied by the novel junction.
  - The percentage of samples of a given tissue in which the novel junction was used, as defined by ≥ 1 novel junction read in the sample.
  - The mean number of novel junction reads across all samples from a given tissue in which the novel junction was detected.
  - The distance in base pairs between the genomic position of the unannotated splice site of the novel junction and the annotated acceptor/donor splice site of its paired annotated intron.
  - Whether the novel junction is capable of causing a frameshift. This value has been calculated by obtaining the modulo 3 of the distance in base pairs lying between the genomic position of the unannotated splice site of the novel junction and the acceptor/donor splice site of its paired annotated intron. When the reminder of this division by 3 (i.e. modulo 3) returns a value of 1 or 2, it indicates that the excision of the novel intron would be capable of causing a frameshift.
  - The strength of the 5’ and 3’ splicing signals implied by the novel junction, as calculated by the MaxEntScan (24) score algorithm.

### Web interface implementation

A ShinyApp website was built and it uses the data stored in the IntroVerse SQLite database. The IntroVerse website was deployed using a Linux-based Apache/2.4.6 Web server running on a Docker container version 18.09.0 over a CentOS Linux release 7.5.1804 (Core) server. ShinyProxy (https://www.shinyproxy.io/) was used as a proxy to allow multiple users to access IntroVerse simultaneously and to provide a fully isolated workspace per R session. Different in-house R scripts (DOI:10.5281/zenodo.6869186) were used to query the IntroVerse SQLite object from the IntroVerse Shiny app and return the information requested within a user-friendly format. Visualisation of the novel junctions as well as the mis-splicing activity occurring within the MANE Select transcript of a particular gene was made available through the use of the R package ggtranscript (35) version 0.99.9. The IntroVerse website was built using numerous client-side languages (HTML, CSS, JavaScript, JQuery) and libraries (JQuery.js, DataTable.js), as well as the Bootstrap version 4 framework (https://getbootstrap.com/), which helped to enhance the look and feel of the user-interface and the user experience by supporting multiple devices, browsers and screen sizes. The IntroVerse website does not require registration or login for access and is freely available for research use and data download at https://rytenlab.com/browser/app/introverse. Finally, IntroVerse can also be downloaded as a docker image from https://hub.docker.com/r/soniaruiz/introverse.

## RESULTS

### Database content and statistics

We analysed 17,510 RNA-sequencing samples across 54 human tissues from GTEx v8 (22) with a median of 248 samples per tissue (ranging between 4 and 805 samples). All the split reads obtained from this dataset were annotated to the Ensembl v105 reference transcriptome. Of the split reads that passed the quality-control criteria (Figure 1C), we identified a total of 332,571 annotated introns of which 294,050 had evidence of at least one type of mis-splicing event, namely the use of a novel donor or novel acceptor junction in at least one of the 17,510 samples studied. The remaining 38,521 annotated introns corresponding to 14,395 genes within the database (44.06% of all genes analysed) had no evidence of mis-splicing across all tissues and samples processed (Figure 2B). Interestingly, of these 14,395 genes, we found that 10,081 were protein-coding and 3,584 were lncRNAs (with the remaining 730 being a mixture of other gene biotypes). Given that relatively few lncRNAs have known biological functions, this finding might suggest that IntroVerse could be used to identify the most important genes of this type. In fact, this view is supported by the finding that introns within lncRNA genes are more frequently mis-spliced at both the donor (Wilcoxon rank sum test with continuity correction, effect-size = 2.8e-2, *P-*value < 2.2e−16) and acceptor splice sites (Wilcoxon rank sum test with continuity correction, effect-size = 3.03e-2, *P-*value < 2.2e−16) than introns within protein-coding genes even after controlling for differences in read coverage (Supplementary Figure S2). Therefore, when we identify lncRNAs which are perfectly spliced this could indicate a specific requirement for efficient degradation of mis-splicing events at this locus. With this in mind, we noted that the mouse homologue of the gene, *FENDRR*, a lncRNA containing multiple introns with no evidence of mis-splicing across samples from all tissues analysed, when knocked out results in postnatal lethality with defects in the lung, gastrointestinal tract and heart in *Fendrr−/−* neonates (36). Furthermore, since its essential role in mouse development was identified, *FENDRR* has also been implicated in multiple human cancers, supporting the utility of IntroVerse in prioritising lncRNAs for investigation (37).

IntroVerse includes information on 1,950,821 novel donor and 2,728,653 novel acceptor junctions, which are confidently paired with the 294,050 annotated introns presenting evidence of at least one type of mis-splicing event across all tissues (Figure 2B). To summarise this data and provide a measure of mis-splicing across each annotated intron, we provide users with the measures, $MS{R_D}$ and $MS{R_A}$. Focusing on samples from frontal cortex tissue, the median $MS{R_D}$ corresponded to 5e-4 [0−0.99], whereas the median $MS{R_A}$ was 1.3e-3 [0−0.99], though it is noteworthy that in both cases the modal values were zero indicating that splicing is largely accurate. Considering all tissues, the median $MS{R_D}$ was 8e-4 and the median $MS{R_A}$ was 1.7e-3, indicating that mis-splicing at the acceptor splice site is more common than that at the donor.

### Web interface and usage

IntroVerse is a user-friendly and comprehensive website that allows users to browse and explore the characteristics of 332,571 annotated introns belonging to 32,669 different genes. The IntroVerse website consists of three major modules that allow users to (i) obtain information on the key characteristics and splicing accuracy of annotated introns across samples within each of the 54 GTEx v8 tissues, (ii) obtain information on the overall usage of novel junctions associated with an annotated intron of interest at both the donor and acceptor sites separately, and across each of the 54 GTEx tissues, and iii) visualise novel junctions and the rate of mis-splicing at donor and acceptor splice sites of annotated introns within the Matched Annotation from NCBI and EMBL-EBI (MANE) Select transcript (34) of a selected gene, and display it across all samples from a single or multiple tissues.

#### Intron search

**Figure 4.**
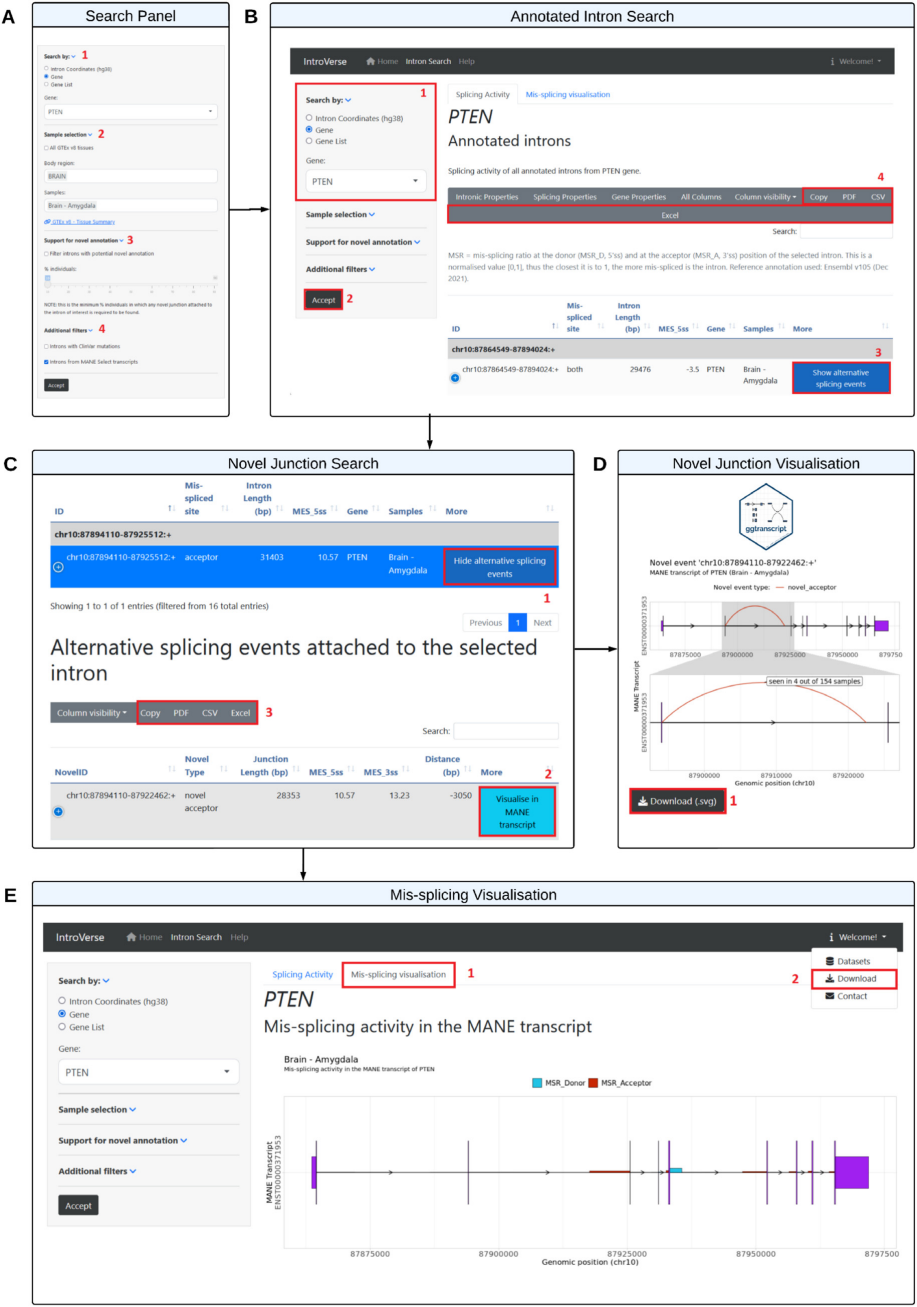
Basic functions of the IntroVerse web interface. (**A**) Overview of the main Search Panel available on the user interface of IntroVerse. (**B**) The displayed columns of the intron table correspond to: (i) ID, the unique identifier of the intron; (ii) Mis-spliced site, the splice site of the intron that has been mis-spliced if any; (iii) Intron Length, which represents the length of the intron in base pairs (bp); (iv) MES_5ss, the strength of the 5' (i.e. donor) splicing signal through the use of the MaxEntScan score algorithm; (v) Gene, the gene to which the intron belongs to; (vi) Samples, cluster of samples in which the intron search has been performed; (vii) More, button ‘Show Alternative Splicing Events’ to show the mis-splicing activity attached to the intron. (**C**) The columns of the novel junction table correspond to: (i) NovelID, the unique identifier of the novel junction; (ii) Novel Type, type of novel event, classified as ‘novel donor’ or ‘novel acceptor’; (iii) Junction Length (bp), implied intron length of the novel junction in base pairs; (iv) MES_5ss, the strength of the 5' (i.e. donor) splicing signal through the use of the MaxEntScan score algorithm; (v) MES_3ss, the strength of the 3' (i.e. acceptor) splicing signal through the use of the MaxEntScan score algorithm; (vi) Distance (bp), distance in base pairs between the novel junction and the paired annotated intron; (vii) More, button ‘Visualise in MANE transcript’ to display the graphical representation of the novel junction within the MANE transcript of the gene. (**D**) Visualisation of a novel donor event within the MANE transcript of a selected gene. Graphs can be downloaded in SVG format. (**E**) Visualisation of the mis-splicing activity occurring in all annotated introns within the MANE transcript of the selected gene. By clicking on the tab ‘Download’, it is possible to download the complete IntroVerse SQLite database.

To search for an annotated intron in IntroVerse within a tissue of interest, users can query the database by (i) introducing the coordinates of an annotated intron of interest (‘*by intron coordinates’*), (ii) selecting a gene symbol or gene ID from the dropdown list provided (*‘by gene’*) and (iii) uploading a gene list of interest (*‘by gene list’*) (Figure 4A-1, Figure 4B-1). The latter requires the user to upload a *.csv* file containing one single column with headers and a gene symbol or gene Ensembl ID per row. An example gene list is also provided. In all three cases, the information returned by IntroVerse consists of a table in which every entry represents an annotated intron in the reference transcriptome (Ensembl v105), with columns providing a range of key features about the annotated intron (see Materials and Methods, *Database generation*) and an additional blue button to show the mis-splicing activity attached to the intron (Figure 4B-3).

IntroVerse also provides an advanced search section which enables the user to refine the query further. First, the *‘Sample Selection’* section (Figure 4A-2) allows the user to determine whether the annotated intron search should be done across one single tissue, multiple tissues or *‘All tissues’*. This latter will produce an intron query across all 17,510 samples from the 54 human GTEx tissues processed. Second, the *‘Support for novel annotation’* section (Figure 4A-3), contains a checkbox and a numeric range that filters the results to only show the annotated introns associated with at least one alternative splicing event shared across the minimum number of samples selected through the numeric range. This search is based on the hypothesis that for any novel junction to be indicative of the presence of a novel stable transcript (rather than mis-splicing), it should be shared across a high number of unrelated individuals. More specifically, we consider mis-splicing to be a random process making the likelihood of excising the same novel junction with the identical genomic positions across multiple unrelated individuals very low. Consequently, a specific novel junction that is detected across a high number of individuals (e.g. ≥ 90%) is more likely to be generated through a deliberate (non-random) process and to be of biological interest. Third, the *‘AdditionalFilters’* section (Figure 4A-4), provides two search refinements: (i) the *‘Introns with ClinVar mutations’* checkbox will only retrieve annotated introns that have been reported to contain at least one pathogenic or likely pathogenic splicing variant as defined within the ClinVar archive (38) and (ii) the *‘Introns from MANE Select transcripts’* checkbox will only report the introns from genes with a protein-coding transcript classified as MANE (34) in the reference annotation.

#### Mis-splicing search

All alternative splicing events that are associated with an annotated intron of interest can be retrieved by clicking the blue button *‘Show Alternative Splicing Events’* located at the end of every row of the annotated Intron table (Figure 4B-3, Figure 4C-1). Clicking this button will automatically hide all rows except those selected and will result in the display of an additional table consisting of all alternative splicing events paired with the annotated intron of interest. In this second table, every entry represents a unique alternative splicing as identified through novel junctions. The columns provide key information about the novel event itself (see Materials and Methods, *Database generation*), and an additional button *‘Visualise in MANE transcript’* (Figure 4C-2) which when clicked will trigger a popup window with the graphical representation of the novel junction within the MANE transcript of the gene (Figure 4D). This visualisation will highlight the related novel donor (blue) or novel acceptor junctions (red) within the gene's MANE (34) transcript.

#### Gene mis-splicing visualisation

IntroVerse provides the option to visualise mis-splicing rates across all annotated introns contained within the MANE transcript of a gene of interest (Figure 4E-1) in a given tissue. The mis-splicing rate as defined by the $MS{R_D}$ and the $MS{R_A}$ values are represented by blue and red vertical bars respectively located at the 5’ splice ($MS{R_D}$) or 3’ splice sites ($MS{R_A}$). Since $MS{R_D}$ and $MS{R_A}$ are normalised to range between a value of 0 and 1, the higher the vertical bar displayed, the higher the proportion of novel junction reads paired to the annotated intron at that splice site. This visualisation allows the user to quickly identify annotated introns within the MANE transcript that are most frequently mis-spliced across samples of single or multiple tissues, with the latter enabling the identification of tissue-specific mis-splicing activity.

### Data download and visualisation

IntroVerse provides functionality for both data download and visualisation purposes. Data tables containing annotated introns and novel junctions can be fully downloaded in PDF, CSV, Excel and Text formats (Figure 4B-4, Figure 4C-3). Depending on the table selected, every entry will represent either an annotated intron or a novel junction, and the columns will contain key information about each feature (see Materials and Methods, *Database generation*) with the user being able to select only a subset of features as required. Data visualisation is provided for novel junctions as well as for mis-splicing activity across all annotated introns within the MANE transcript of a selected gene. Visualisation for novel junctions can be downloaded in SVG format (Figure 4D-1). The complete SQLite database can be downloaded directly from the user interface of IntroVerse along with other useful resources such as the SQL schema and a set of handy SQL queries (Figure 4E-2). The IntroVerse database has been carefully designed to be highly efficient such that the entire database currently occupies less than 3GB.

## APPLICATIONS

IntroVerse has multiple use cases and some of them are listed below:

1. The assessment of background levels of mis-splicing amongst control individuals within introns thought to be mis-spliced in disease states. We found that introns containing pathogenic or likely pathogenic splice site mutations (as defined within ClinVar, https://www.ncbi.nlm.nih.gov/variation/view) have significantly lower $MS{R_D}$ (Wilcoxon rank sum test with continuity correction, effect-size = 5.78e-2, *P-*value < 2.2e−16) and $MS{R_A}$ (Wilcoxon rank sum test with continuity correction, effect-size = 6.74e-2, *P-*value < 2.2e−16) values across all tissues than other annotated introns not containing any ClinVar variant after controlling for mean read coverage (Supplementary Figure S3). This suggests that, in control individuals, annotated introns containing pathogenic variants are spliced with particularly high accuracy.
2. Identification of novel transcripts of known genes and their tissue-specific usage: Using samples from frontal cortex tissue, we detected 2,048 novel junctions corresponding to 1,420 genes which are used in > 80% of samples (*N* = 167), suggesting the existence of incomplete annotation at these specific sites. Importantly, we found that the discovery of such sites was only weakly correlated with read coverage over the annotated intron (*R*^2^ = 3.7e-2, *P-*value < 2e−16, Supplementary Figure S1). Furthermore, by using the filter *‘Support for novel annotation’*, which is available on the main user interface of IntroVerse, we noted that of all green genes (*N* = 121) causally implicated in hereditary ataxia (as defined in PanelApp Version 1.303, https://panelapp.genomicsengland.co.uk/), 17% (*N* = 21) contain novel junctions which are used in ≥ 80% of cerebellar samples (*N* = 217).
3. Integration of short- and long-read RNA-sequencing data to support novel transcript discovery: Using untargeted long-read RNA-sequencing data provided by the ENCODE project and relating to N = 16 samples of the human heart (Supplementary Table S2), we identified all novel junctions absent from Ensembl v105. Of the 611 unique novel junctions found across all sixteen ENCODE samples, we found that 349 were also detected in GTEx v8 left ventricle and atrial appendage heart tissues as stored in IntroVerse. Furthermore, these 342 novel junctions were detected in a median of 353 left ventricle and atrial appendage GTEx samples suggesting that the full-length transcripts from which they are derived are likely to be novel transcripts of known genes.

## DISCUSSION

IntroVerse provides quality-controlled information on the splicing of 332,571 annotated introns from 32,669 different genes across 17,510 RNA-sequencing samples and 54 human control tissues. IntroVerse has two unique features: (i) it provides a complete and unfiltered catalogue of novel junctions that have been carefully processed and quality-controlled and (ii) each novel junction has been assigned to a specific annotated intron, which provides a hierarchical structure allowing the user to start by browsing a gene or genomic position of interest and to end with an exploration of the complete catalogue of alternative events occurring at that locus. In addition, IntroVerse allows users to browse, download and compare key genomic parameters either across introns of a particular gene and sample of interest or across multiple genes, samples and human tissues. Finally, to the best of our knowledge, IntroVerse is the only database that enables the visualisation of alternative splicing events by i) displaying individual events in the context of the gene's MANE transcript and ii) the overall frequency of alternative splicing of each annotated intron of the gene's MANE transcript.

Future versions of this database will include additional human RNA-sequencing data sets generated from individuals with disease, in addition to controls. More specifically, we will include data provided by the BrainSEQ Consortium Phase I and II projects (39–41), which have generated RNA-sequencing data from more than 700 human brain samples across the lifespan and derived from individuals with major neuropsychiatric disorders. With the increasing availability of untargeted long-read RNA-sequencing data, we will also expand the annotation of novel junctions within IntroVerse to flag when a junction is also identified within public long-read RNA-sequencing data and the related full-length transcript/s. These future updates will increase the value of IntroVerse and broaden its applications.

Thus, IntroVerse provides users with structured data on intron usage across a wide range of human tissues which can be used in a range of downstream analyses to improve our understanding of the role of splicing in health and disease.

## DATA AVAILABILITY

IntroVerse is a user-friendly and comprehensive website that is fed from an SQLite database containing the splicing activity of 332,571 annotated introns found across 17,510 RNA-sequencing samples from 54 control tissues. The complete SQLite database is available to be downloaded directly from the user interface of IntroVerse, as it has been carefully designed to be highly efficient such that the entire database currently only occupies less than 3GB.

IntroVerse is available for data-querying and download purposes at https://rytenlab.com/browser/app/introverse. Multiple in-house R scripts have been created to query the IntroVerse SQLite object from the IntroVerse Shiny app, as well as to build the latter. These scripts are freely available on GitHub (DOI:10.5281/zenodo.6869186).

Finally, IntroVerse is also available in docker formatting at https://hub.docker.com/r/soniaruiz/introverse.

## Supplementary Material

gkac1056_Supplemental_File
