# Supplementary material for: IntroVerse: a comprehensive database of introns across human tissues
Source: Nucleic Acids Res. 2022 Nov 18;51(D1):D167–78. doi: 10.1093/nar/gkac1056 (PMC9825543; doi:10.1093/nar/gkac1056)
Supplement: gkac1056_Supplemental_File [file gkac1056_supplemental_file.pdf]

Supplementary Figures

Supplementary Figure1.

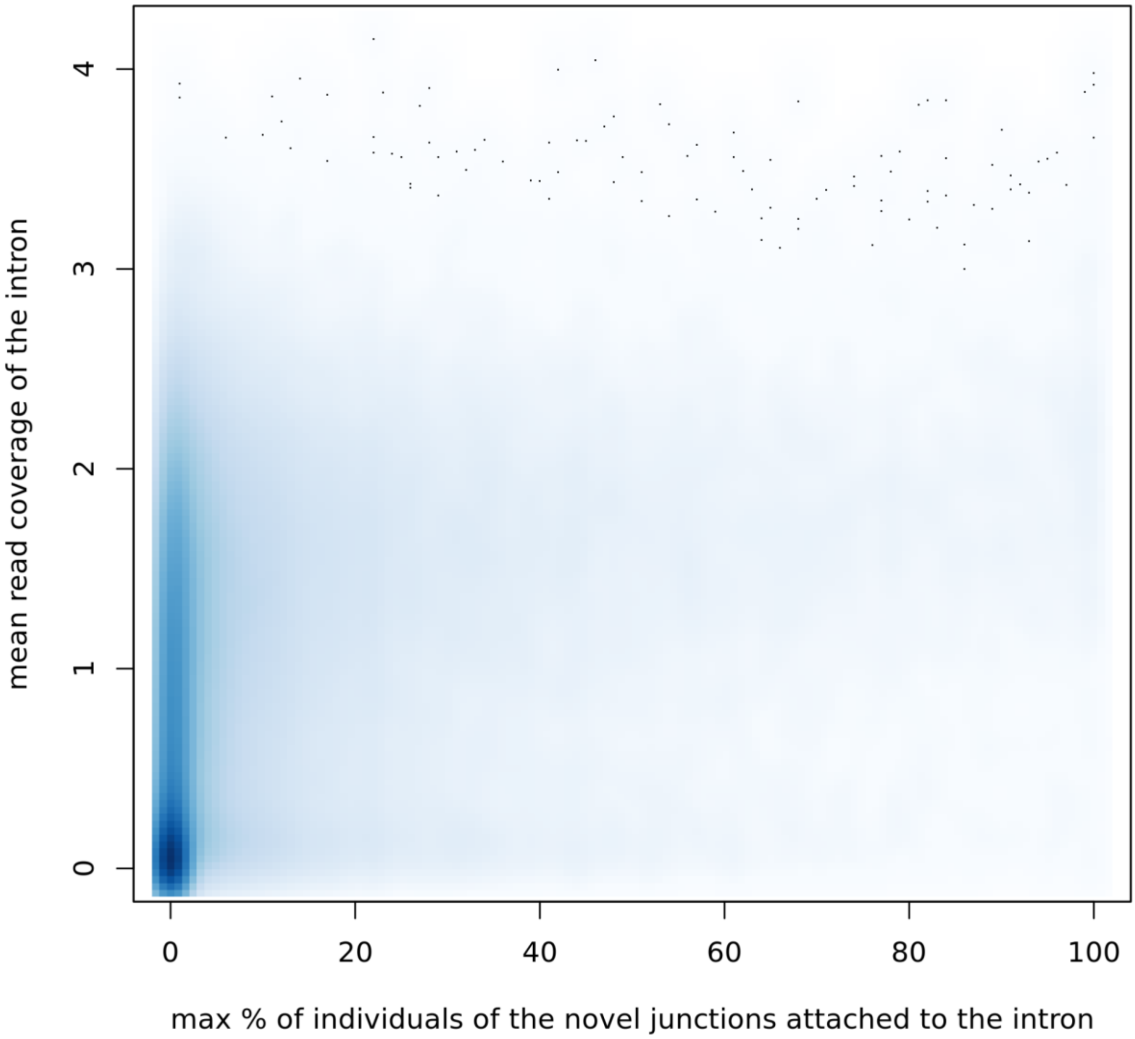

Scatter plot to display the relationship between the mean read coverage of each annotated intron in frontal cortex tissue (N = 239,953) and the maximum percentage of individuals sharing any of its novel junctions. For appropriate resolution and interpretability purposes, a logarithm in base 10 of the mean read coverage was calculated and displayed within the figure. A Pearson Correlation analysis between the two variables showed that the % of individuals sharing a particular novel junction was only weakly correlated with the mean read coverage of the annotated intron (R^2=0.037, pval<2e-16).

Supplementary Figure2.

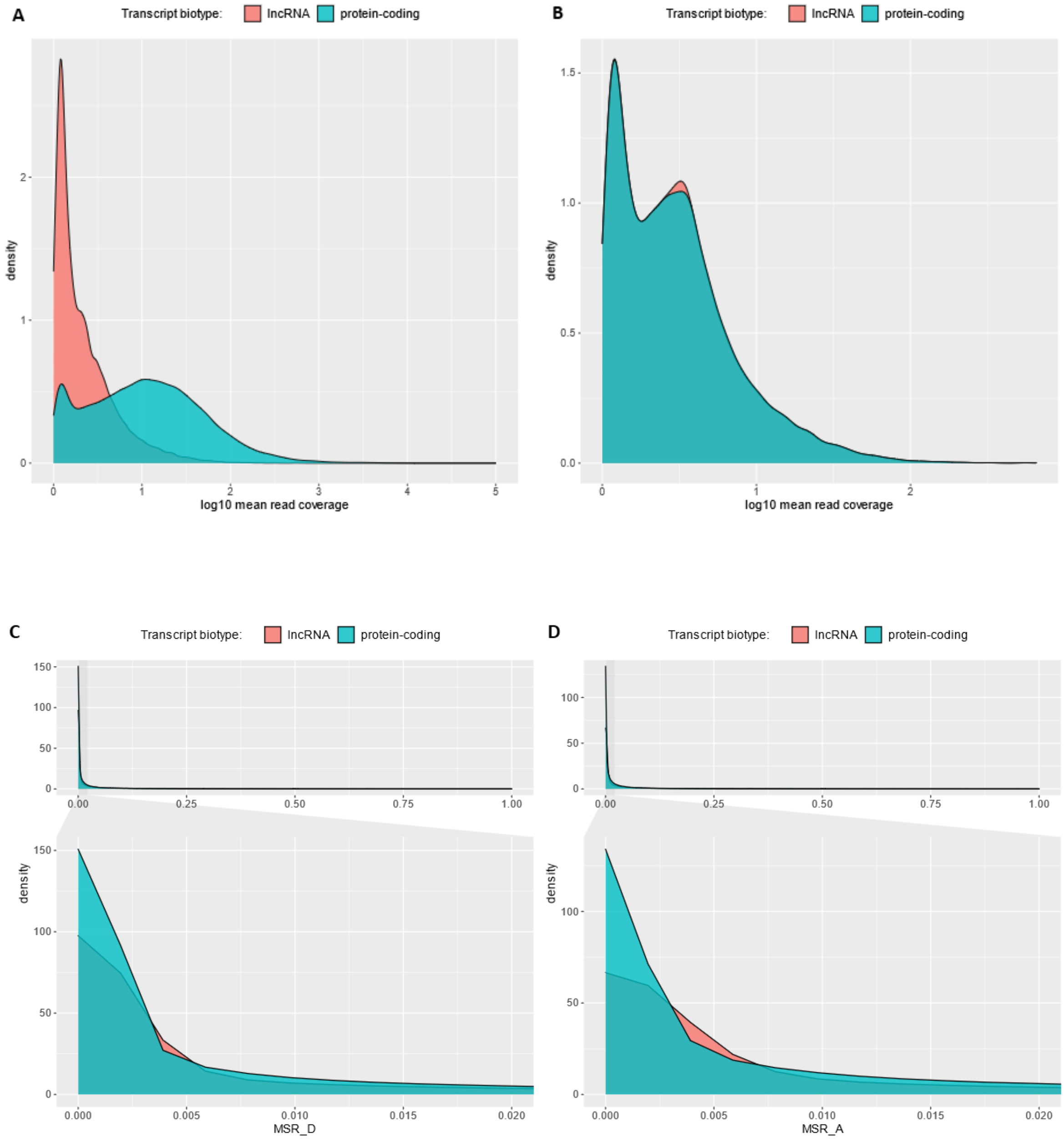

Overview of the subsampling process performed over the introns from lncRNA transcripts and introns from protein-coding transcripts as stored on IntroVerse, to control by mean read coverage. (A). Mean read coverage of the annotated introns that only belong to protein-coding transcripts (N=74,964, represented in green) across the samples from all the tissues studied and the annotated introns which are only part of lncRNA transcripts (N=52,191, represented in red). The mean read coverage figure was calculated at the intron level by dividing the cumulative number of reads that each intron presented by the total number of samples in which each intron was found. For

appropriate resolution and interpretability purposes, a logarithm in base 10 of the mean read coverage was calculated prior to display it within the figure. The differences shown in these two distributions suggest that lncRNA transcripts tend to be expressed at lower levels than protein-coding transcripts. **(B).** Mean read coverage of the annotated introns from lncRNA (in red) and protein-coding (in green) transcripts after subsampling the annotated introns to control for mean read coverage. The subsampling was performed by pairing the introns depending on their mean read coverage similarity. The maximum mean read coverage difference allowed corresponded to 0.005 (R function used: `matchit()`, from the MatchIt R package, Version 4.0.0). This subsampling reduced both distributions to a total of N=29,933 lncRNA and N=29,933 protein-coding introns, respectively. **(C)** Distribution of the MSR_D values of the introns from lncRNA transcripts versus introns from protein-coding transcripts after subsampling both distributions to control by mean read coverage. This analysis shows that introns from lncRNA transcripts are more frequently mis-spliced at the donor splice site than introns from protein-coding transcripts (Wilcoxon rank sum test with continuity correction, effect-size=0.028, p-value < 2.2e-16). **(D)** Distribution of the MSR_A values of the introns from lncRNA transcripts versus introns from protein-coding transcripts after subsampling both distributions to control by mean read coverage. This analysis shows that introns from lncRNA transcripts are more frequently mis-spliced at the acceptor splice site than introns from protein-coding transcripts (Wilcoxon rank sum test with continuity correction, effect-size=0.0303, p-value < 2.2e-16).

Supplementary Figure3.

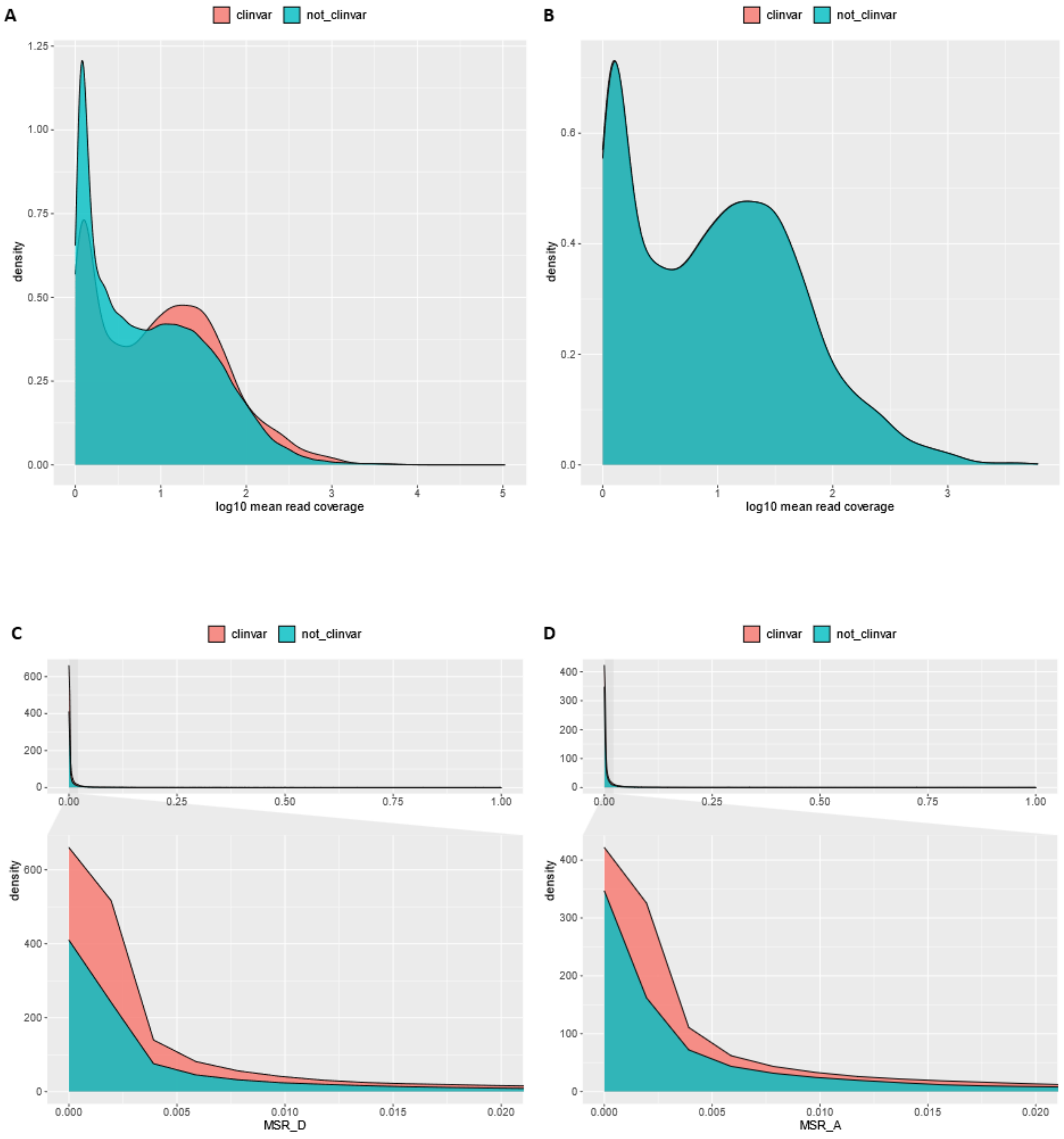

Overview of the subsampling process performed over the introns containing/not-containing likely pathogenic/pathogenic splicing mutations, as stored on IntroVerse, to control by mean read coverage. (A). Mean read coverage across the samples from all tissues of the annotated introns in which likely pathogenic/pathogenic splicing mutations are located (N=4,852, represented in red) as reported by ClinVar, and the introns that do not contain mutations of this type (N=327,719, represented in green). The mean read coverage figure was calculated at the intron level by dividing the cumulative number of reads that each intron presented by the total number of samples in which each annotated intron was found. For appropriate resolution and interpretability purposes, a logarithm in base 10 of the mean read coverage was then calculated and displayed within the figure. This analysis suggested that mean read coverage differs between introns containing and not containing pathogenic/likely pathogenic mutations, and read coverage was higher for annotated

introns reported to contain splicing mutations. **(B).** Subsampled distribution of mean read coverage of the introns containing/not containing ClinVar mutations. The subsampling was performed by pairing the introns depending on their mean read coverage similarity (i.e. with a maximum mean read coverage difference of 0.005, `matchit()` function from the MatchIt R package, Version 4.0.0). This subsampling approach resulted in the generation of two distributions containing a total of N=4,852 matching number of annotated introns. **(C)** Distribution of the MSR_D values of the introns with ClinVar mutations versus introns without ClinVar mutations after subsampling both distributions of introns to control by mean read coverage. This analysis shows that introns with ClinVar mutations are less frequently mis-spliced at the donor splice site than introns without ClinVar mutations (Wilcoxon rank sum test with continuity correction, effect-size=0.0578, p-value<2.2e-16). **(D)** Distribution of the MSR_A values of the introns with ClinVar mutations versus introns without ClinVar mutations after subsampling both distributions of introns to control by mean read coverage. This analysis shows that introns with ClinVar mutations are less frequently mis-spliced at the acceptor splice site than introns not containing ClinVar mutations (Wilcoxon rank sum test with continuity correction, effect-size=0.0674, p-value < 2.2e-16).

Supplementary Tables

Supplementary Table1.

**GTEx v8 tissue**	**Total number of unique annotated introns**
Adipose - Subcutaneous	250,603
Adipose - Visceral (Omentum)	249,041
Adrenal Gland	238,319
Artery - Aorta	243,625
Artery - Coronary	237,879
Artery - Tibial	247,608
Bladder	208,538
Brain - Amygdala	236,777
Brain - Anterior cingulate cortex (BA24)	238,783

Brain - Caudate (basal ganglia)	241,177
Brain - Cerebellar Hemisphere	236,037
Brain - Cerebellum	238,889
Brain - Cortex	243,222
Brain - Frontal Cortex (BA9)	239,953
Brain - Hippocampus	239,355
Brain - Hypothalamus	240,971
Brain - Nucleus accumbens (basal ganglia)	241,756
Brain - Putamen (basal ganglia)	237,829
Brain - Spinal cord (cervical c-1)	235,181
Brain - Substantia nigra	234,382

Breast - Mammary Tissue	251,865
Cells - Cultured fibroblasts	239,725
Cells - EBV-transformed lymphocytes	228,192
Cervix - Ectocervix	196,842
Cervix - Endocervix	201,067
Colon - Sigmoid	243,628
Colon - Transverse	245,990
Esophagus - Gastroesophageal Junction	241,648
Esophagus - Mucosa	245,631
Esophagus - Muscularis	247,842
Fallopian Tube	200,885

Heart - Atrial Appendage	242,644
Heart - Left Ventricle	239,029
Kidney - Cortex	228,906
Kidney - Medulla	187,551
Liver	233,329
Lung	251,424
Minor Salivary Gland	234,819
Muscle - Skeletal	250,631
Nerve - Tibial	250,779
Ovary	234,369
Pancreas	238,577

Pituitary	247,107
Prostate	245,994
Skin - Not Sun Exposed (Suprapubic)	250,923
Skin - Sun Exposed (Lower leg)	252,130
Small Intestine - Terminal Ileum	239,568
Spleen	236,722
Stomach	243,275
Testis	279,156
Thyroid	253,440
Uterus	230,226
Vagina	233,555

Whole Blood	240,487

Summary of the total number of unique annotated introns per tissue as stored in IntroVerse.

Supplementary Table2.

**ENCODE tissue**	**ENCODE sample ID**	**DOI**	**Donor**
left_cardiac_atrium	ENCSR424QFN	doi:10.17989/ENCSR424QFN	female adult (59 years)
left_ventricle	ENCSR194YUY	doi:10.17989/ENCSR194YUY	female adult (53 years)
left_ventricle	ENCSR575LWI	doi:10.17989/ENCSR575LWI	female adult (59 years)
left_ventricle	ENCSR700XDQ	doi:10.17989/ENCSR700XDQ	female adult (46 years)
left_ventricle	ENCSR994YZY	doi:10.17989/ENCSR994YZY	male adult (40 years)

left_ventricle_myocard_sup	ENCSR777CCI	doi:10.17989/ENCSR777CCI	male adult (60 years)
left_ventricle_myocardium_inferior	ENCSR786FLO	doi:10.17989/ENCSR786FLO	male adult (60 years)
right_cardiac_atrium	ENCSR435UUS	doi:10.17989/ENCSR435UUS	male adult (60 years)
right_cardiac_atrium	ENCSR514YQN	doi:10.17989/ENCSR514YQN	female adult (46 years)
right_cardiac_atrium	ENCSR553SVP	doi:10.17989/ENCSR553SVP	female adult (59 years)
right_cardiac_atrium	ENCSR728TXV	doi:10.17989/ENCSR728TXV	male adult (40 years)
right_ventricle	ENCSR329ZQG	doi:10.17989/ENCSR329ZQG	female adult (59 years)

right_ventricle	ENCSR782LGT	doi:10.17989/ENCSR782LGT	female adult (46 years)
right_ventricle	ENCSR984OAE	doi:10.17989/ENCSR984OAE	male adult (40 years)
right_ventricle_myocardium_inferior	ENCSR591OZR	doi:10.17989/ENCSR591OZR	male adult (60 years)
right_ventricle_myocardium_inferior	ENCSR899GAP	doi:10.17989/ENCSR899GAP	male adult (60 years)

Details of the sixteen long read RNA-seq ENCODE samples analysed. All these samples had been sequenced following untargeted long-read RNA-sequencing protocol.
